# Intelligent prediction of Alzheimer’s disease via improved multifeature squeeze-and-excitation-dilated residual network

**DOI:** 10.1038/s41598-024-62712-w

**Published:** 2024-05-25

**Authors:** Zengbei Yuan, Xinlin Li, Zezhou Hao, Zhixian Tang, Xufeng Yao, Tao Wu

**Affiliations:** 1 College of Medical Imaging, Jiading District Central Hospital Affiliated Shanghai University of Medicine and Health Sciences, Shanghai, 201318 China; 2 School of Health Science and Engineering, University of Shanghai for Science and Technology, Shanghai, 200093 China

**Keywords:** Alzheimer's disease, Machine learning

## Abstract

This study aimed to address the issue of larger prediction errors existing in intelligent predictive tasks related to Alzheimer’s disease (AD). A cohort of 487 enrolled participants was categorized into three groups: normal control (138 individuals), mild cognitive impairment (238 patients), and AD (111 patients) in this study. An improved multifeature squeeze-and-excitation-dilated residual network (MFSE-DRN) was proposed for two important AD predictions: clinical scores and conversion probability. The model was characterized as three modules: squeeze-and-excitation-dilated residual block (SE-DRB), multifusion pooling (MF-Pool), and multimodal feature fusion. To assess its performance, the proposed model was compared with two other novel models: ranking convolutional neural network (RCNN) and 3D vision geometrical group network (3D-VGGNet). Our method showed the best performance in the two AD predicted tasks. For the clinical scores prediction, the root-mean-square errors (RMSEs) and mean absolute errors (MAEs) of mini-mental state examination (MMSE) and AD assessment scale–cognitive 11-item (ADAS-11) were 1.97, 1.46 and 4.20, 3.19 within 6 months; 2.48, 1.69 and 4.81, 3.44 within 12 months; 2.67, 1.86 and 5.81, 3.83 within 24 months; 3.02, 2.03 and 5.09, 3.43 within 36 months, respectively. At the AD conversion probability prediction, the prediction accuracies within 12, 24, and 36 months reached to 88.0, 85.5, and 88.4%, respectively. The AD predication would play a great role in clinical applications.

## Introduction

Alzheimer’s disease (AD) is a type of common neurodegenerative disease that generally accompanies with progressive impairments in human cognition, behavior, and analytical skills^1^. AD has a prevalence of 4.4% in people older than 65 years, and its treatment cost has exceeded 300 billion dollars worldwide^2,3^. Early prediction of AD could effectively help to intervene the progression in time and improve the outcome of treatment^4^. Recently, the intelligent prediction of AD via deep learning (DL) algorithms has brought new opportunities for early diagnosis of AD^5^.

During the progression of AD, mild cognitive impairment (MCI) is regarded as the critical transition period from normal to AD. Patients with MCI have been reported to be 10 times more likely than normal individuals to progress to AD, and about 70% of MCI patients deteriorate to AD within 5 years. MCI is generally classified into two subtypes: progressive MCI (pMCI) and stable MCI (sMCI), based on the progression of the disease. Timely intervention in the MCI stage has shown potential in delaying the prognosis of the disease and significantly improve the prognosis of patients. Therefore, accurate prediction of AD conversion probability at the MCI stages has garnered significant interest among clinical researchers^6^.

In clinical practice, the diagnostic methods of AD mainly include neuropsychological testing, neuroimaging, genetic testing, and biomarker testing. A series of neuropsychological tests, such as mini-mental state examination (MMSE), AD assessment scale-cognitive 11-item (ADAS-11), and so forth, played a crucial role in AD diagnosis and provided an in-depth insight into the state of cognitive functioning^7,8^. The neuroimaging modalities of magnetic resonance imaging (MRI) and positron emission tomography (PET) have been widely used in evaluating AD. For accurate quantitative evaluation of the structural changes in the brain along with AD progress, structural magnetic resonance imaging (sMRI) has become the most popular imaging technique in AD evaluation^9^. Thus far, several susceptibility genes have been identified for AD include amyloid precursor protein, presenilin 1, presenilin 2, and apolipoprotein E (ApoE). Among these genes, ApoE is closely correlated with AD occurrence. In addition, the biomarker testing was applied to monitor the state of AD progression by measuring the concentration of specific antibodies and proteins, including Aβ1-41, Aβ42/Aβ40, total tau protein (t-tau), and hyperphosphorylated tau protein (p-tau) in the cerebrospinal fluid or blood^10,11^. Therefore, with the multimodality clinical data, the deduced features of sMRI, genes, biomarkers, and clinical scores have been used for AD predication^12–16^.

In the literature, traditional ML and DL algorithms have made great progress in the AD classification. Once, Arafa et al.^17^ used a traditional CNN model for the classification of mild-dementia and non-dementia with an accuracy of 99.99%. And Fathi et al.^18^ applied six CNN classifiers to form an integrated model with up to 93.92% accuracy. Then, Helaly et al.^19^ used the traditional CNN model, the classification accuracies for 2D and 3D data of AD were 93.61 and 95.17%, respectively, and VGG19 models for transfer learning with up to 97% accuracy. Notably, Shankar et al.^20^ developed a novel hierarchical residual attention learning-inspired multistage conjoined twin network (HRAL-CTNN) and got an classification accuracy of 99.97%.

Besides, two crucial AD prediction tasks have been identified: clinical scores and conversion probability. For AD intelligent prediction, alterations in clinical scores and conversion probability at different time periods have been investigated by the traditional machine learning (ML) and DL algorithms.

It was reported that traditional ML algorithms were proposed for predicting clinical scores. Zhou et al.^16^ proposed a convex fused sparse group least absolute shrinkage and selection operator (LASSO) model using MRI and genetic and demographic features to predict clinical scores, and eventually obtained the root-mean-square errors (RMSEs) of 2.737 and 5.678 for MMSE and ADAS within 12 months, respectively. Then, Lei et al.^21^ used MRI and clinical score features to construct a feature selection model of LASSO and correlation entropy to predict MMSE within 18 months, and achieved a mean absolute error (MAE) of 1.74. Again, Tabarestani et al.^22^ applied random forest from PET, MRI, and genetic and clinical score features for MMSE prediction within 24 months, with an RMSE of 3.15.

Notably, the DL models achieved robust performance in clinical scores prediction. Tabarestani et al.^23^ used PET, MRI, clinical scores, and genetic and fluid biomarker features to construct a long short-term memory model to predict MMSE. They obtained an RMSE of 1.97 within 12 months. Liu et al.^24^ proposed a weakly supervised densely connected neural network model using combined features of MRI and clinical scores, and eventually obtained the prediction RMSEs of 3.408 and 7.451 for MMSE and ADAS within 24 months, respectively. Additionally, Zhang et al.^25^ used a sparse linear regression model combined with MRI, PET and clinical scoring features to predict the RMSE of 2.035 within 24 months for MMSE. Furthermore, Liu et al.^26^ proposed a deep multitask multichannel learning (DM2L) model with MRI and demographic features to predict ADAS with a RMSE of 6.2 within 36 months.

For the AD conversion probability prediction, a series of traditional ML algorithms have been addressed. Tangaro et al.^27^ proposed a support vector machine (SVM) based fuzzy class algorithm model using multifeatures of the hippocampal changes and clinical scores, and obtained an accuracy (ACC) of 83.4% for AD conversion probability within 12 months. Moreover, Shu et al.^28^ used an integrated ML model to combine MRI, clinical scores and genetic features, and got a conversion probability within 12 months with an area under the curve (AUC) of 0.814. Lin et al.^29^ implemented an extreme learning machine with MRI, PET, and genetic and biological features to achieve an ACC of 83.8% for predicting AD conversion probability within 24 months. Furthermore, Ezzati et al.^30^ applied an ensemble linear discriminant model using MRI and demographic and genetic features to obtain an ACC of 74.9% within 24 months. Also, Gaser et al.^31^ used a relevance vector machine model using clinical scores, hippocampus, and biomarker features to predict AD conversion probability within 36 months and obtained an ACC of 81%.

Accordingly, many DL algorithms have been proposed for AD conversion probability prediction. Recently, Llano et al.^32^ used MRI and clinical scores to predict the AD conversion probability using a tree-based multivariate model and obtained an AUC of 0.665 within 12 months. More recently, Lee et al.^33^ applied a recurrent neural network model with MRI, demographics, fluid biomarkers, and genetic features for predicting AD conversion probability within 24 months and obtained an ACC of 75%. Spasov et al.^34^ used MRI, clinical scores, and genetic and demographic features to construct a 3D divisible convolutional neural network for AD conversion probability prediction and got an ACC of 86% within 36 months. Then, Lu et al.^35^ used deep neural networks using PET and sMRI features and achieved an ACC of 82.4% within 36 months for AD conversion probability prediction. Overall, the DL models have shown better capability in the two predicted tasks of clinical scores and conversion probability in AD. However, there are still challenges to overcome, as large prediction errors and low accuracy remain a concern.

To resolve these problems, the 3D multifeature squeeze-and-excitation-dilated residual network (MFSE-DRN) was proposed for predicting clinical scores and conversion probability in AD. The MFSE-DRN model incorporates three novel modules: squeeze-and-excitation-dilated residual block (SE-DRB), multifusion pooling (MF-Pool), and multimodal feature fusion. These modules enhance the model’s ability to extract multimodal features while maintaining stability to prevent overfitting. By implementing the MFSE-DRN model, we accomplished longitudinal predictions of clinical scores and conversion probability in AD using baseline data. This approach offers a fresh perspective for early intelligent diagnosis of AD, providing valuable insights for improved patient care and management.

## Results

### Clinical scores prediction

During the ablation experiments, the proposed model showed lower RMSE and MAE values than the control group for MMSE and ADAS-11 score prediction at 6, 12, 24, and 36 months (M06, M12, M24, and M36) (Table 1). As shown in Table 1, even after removing the added modules sequentially, the proposed model exhibited significantly lower RMSE and MAE values than the control group for MMSE and ADAS-11 score prediction at M06, M12, M24, and M36. Thus, the results of the ablation experiment validated the effectiveness of each added module in AD prediction.

**Table 1:** Ablation comparison of clinical scores prediction.

Groups	Indices	MMSE				ADAS-11			
		M06	M12	M24	M36	M06	M12	M24	M36
Without DC, SE and MF-pool	RMSE	2.56	3.11	3.48	3.97	5.73	10.57	8.69	6.00
	MAE	2.12	2.58	2.87	3.06	4.83	9.51	7.53	4.71
Without SE	RMSE	1.91	2.44	3.56	3.12	6.82	6.83	7.47	6.03
	MAE	1.48	1.79	2.65	2.05	6.00	5.61	5.63	4.80
Without MF-pool	RMSE	2.43	2.76	2.95	3.35	4.64	5.88	7.60	5.82
	MAE	2.01	2.14	2.28	2.69	3.67	4.70	5.09	4.17
Without DC	RMSE	2.54	2.63	3.01	3.05	5.46	5.02	5.85	5.24
	MAE	2.11	1.80	2.07	2.04	4.48	3.65	3.87	3.44
Without multi-feature fusion	RMSE	2.70	3.30	3.29	3.03	6.24	7.17	6.98	6.07
	MAE	1.93	2.21	2.12	2.06	4.56	4.90	4.60	4.22
MFSE-DRN	RMSE	1.97	2.48	2.67	3.02	4.20	4.81	5.81	5.09
	MAE	1.46	1.69	1.86	2.03	3.19	3.44	3.83	3.43

MMSE, mini-mental state examination; ADAS-11, AD assessment scale-cognitive 11-item; DC, dilated convolution; SE, squeeze-and-excitation; MF-Pool, multifusion pooling; MFSE-DRN, multifeature squeeze-and-excitation-dilated residual network.

Table 2 shows that the proposed model demonstrated best performance in the two tasks of predicting MMSE and ADAS-11 clinical scores and conversion probability within M06, M12, M24, and M36. In MMSE prediction, the proposed model had the lowest RMSEs of 1.97, 2.48, 2.67, and 3.02 and MAEs of 1.46, 1.69, 1.86, and 2.03 within M06, M12, M24, and M36, respectively. For ADAS-11 prediction, the proposed model had the lowest RMSEs of 4.20, 4.81, 5.81, and 5.09 and MAEs of 3.19, 3.44, 3.83, and 3.43 within M06, M12, M24, and M36, respectively.

**Table 2:** Performance comparison of clinical scores prediction.

Models	Indices	MMSE				ADAS-11			
		M06	M12	M24	M36	M06	M12	M24	M36
RCNN	RMSE	2.79	3.28	3.42	3.25	6.46	7.00	7.02	5.93
	MAE	2.13	2.40	2.55	2.47	4.85	5.04	4.79	4.20
3D-VGGNet	RMSE	3.87	3.86	5.92	7.05	6.37	7.16	6.93	6.83
	MAE	2.94	2.81	4.66	5.05	4.84	5.04	4.53	4.63
MFSE-DRN	RMSE	1.97	2.48	2.67	3.02	4.20	4.81	5.81	5.09
	MAE	1.46	1.69	1.86	2.03	3.19	3.44	3.83	3.43

MMSE, mini-mental state examination; ADAS-11, AD assessment scale-cognitive 11-item; RCNN, ranking convolutional neural network; 3D-VGGNet, 3D vision geometrical group network; MFSE-DRN, multifeature squeeze-and-excitation-dilated residual network; RMSE, root mean square error; MAE, mean absolute error.

### AD conversion probability

**Figure 1:**
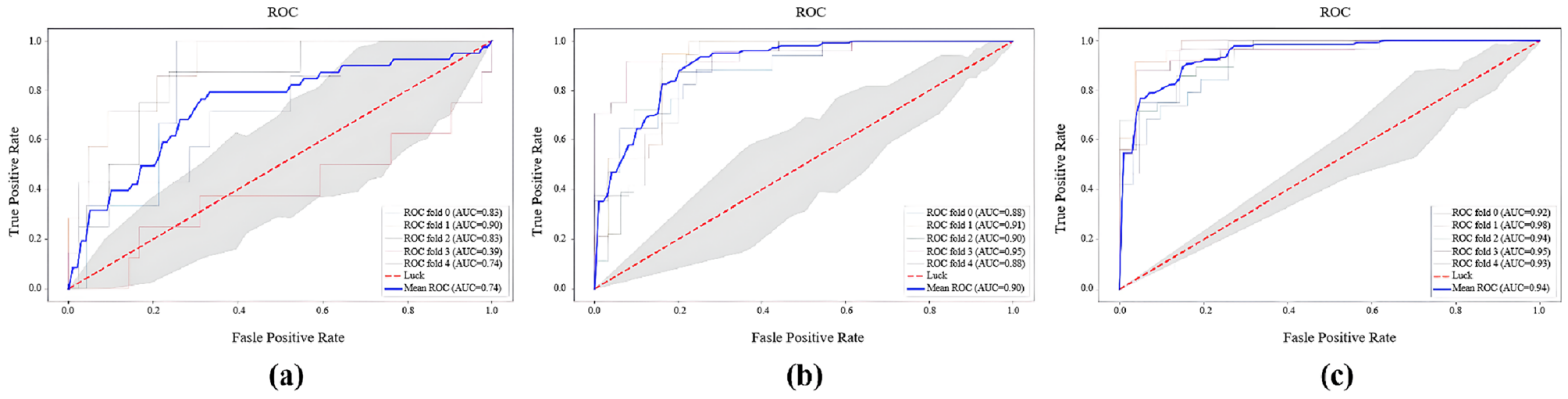
The ROC curves for M12, M24 and M36. (**a**) 12 months; (**b**) 24 months; (**c**) 36 months. ROC: receiver operating characteristic, AUC: area under curve.

According to the ablation experiments, the proposed model obtained the highest predicted ACCs compared to ablation controls within M12, M24, and M36, respectively, as shown in Table 3. As depicted in Table 4, the proposed MFSE-DRN model exhibited superior capability in the AD conversion probability prediction with the highest ACCs of 88.0, 85.5, and 88.4% within M12, M24, and M36. As shown in Fig. 1, the AUCs of the proposed model reached to 0.74, 0.90, and 0.94 for AD conversion probability within M12, M24, and M36, respectively.

**Table 3:** Ablation comparison of AD conversion probability (ACC %).

Groups	M12	M24	M36
Without DC, SE and MF-pool	87.0	85.2	54.7
Without SE	61.0	85.2	87.1
Without MF-pool	87.0	73.0	57.2
Without DC	86.5	67.7	86.1
Without multi-feature fusion	86.7	69.1	71.1
MFSE-DRN	88.0	85.5	88.4

DC, dilated convolution; SE, squeeze-and-excitation; MF-pool, multifusion pooling; MFSE-DRN, multifeature squeeze-and-excitation-dilated residual network.

**Table 4:** Performance comparison of AD conversion probability prediction (%).

Models	M12				M24				M36			
	ACC	PRE	REC	F1	ACC	PRE	REC	F1	ACC	PRE	REC	F1
RCNN	86.7	86.7	100	92.9	76.3	75.0	64.0	65.1	77.9	87.3	65.5	73.7
3D-VGGNet	77.8	90.1	84.5	85.7	74.7	78.0	52.1	60.5	77.9	77.7	77.5	77.5
MFSE-DRN	88.0	88.1	99.5	93.4	85.5	78.8	89.3	82.7	88.4	86.8	89.9	87.7

RCNN, ranking convolutional neural network; 3D-VGGNet, 3D vision geometrical group network; MFSE-DRN, multifeature squeeze-and-excitation-dilated residual network; ACC, accuracy; PRE, precision; REC, recall.

## Discussion

In clinical practice, accurate predictions of clinical scores and conversion probability in AD play a crucial role in understanding the rate of disease progression and tailoring individualized treatment plans. The longitudinal prediction of AD demonstrated significant values in evaluating the progression of AD and preventing its onset. In this study, the proposed model exhibited robust performance in AD predictions of clinical scores and conversion probability. These findings highlight the potential of the model for aiding clinicians in making informed decisions and improving patient outcomes. This is expected to be applied to clinical research.

The combined features of sMRI, genetics, clinical scores, and biomarkers were confirmed to have high sensitivity and specificity for AD. The results showed that the fusion of multiple features ensured that the vital features were acquired during feature extraction and improved the model robustness. It was consistent with the previous findings that various types of data enhanced the model’s ability to learn more complementary information during training^36–38^. Therefore, this study utilized multiple types of baseline data to betterment the effectiveness of the model. Due to the absence of the follow-up data for the CDRSB and RAVLT immediate scores of the participants in the data set, it was worthy of noting that our study did not predict these two clinical scores.

Different from the existing AD prediction models, our proposed model did have substantial innovations in three aspects. Firstly, the construction of the DRB could extract internal core characteristics of sMRI facilitating efficient model^39^. Secondly, the incorporation of the SE structure enhanced the DRB’s ability to accurately capture feature correlations and maintain model stability when handing high-dimensional data^40^. Thirdly, the inclusion of both max pooling and average pooling layers in the multifusion pooling (MF-Pool) block enhanced the model’s capability to extract and delineate nonlinear features while also reducing dimensionality. This enabled the neural network to effectively extract high-dimensional features. As shown in previous studies, the use of multimodal features provides abundant feature information to precisely reflect the pathological process of AD and thus enhances the performance of the model to accurately predict AD^41–43^.

Our experimental results displayed that the prediction RMSEs and MAEs of clinical scores gradually increased along with the follow-up time points. This means that the prediction of clinical scores with typical brain morphology is more accurate in the early stages. This is because the hippocampal and internal olfactory cortical brain regions show a marked tendency to atrophy in the early stages of AD^16^. Similarly, it was validated that those cortices were highly correlated with the MMSE and ADAS in our experiment. Subsequently, the prediction error increased along with the trend of slow cognitive decline in the later stages of AD^24,44^. According to the AD conversion probability, there existed obvious trends of increased ACCs and AUCs along with the varied predicted time points. It was due to the fact that the significant changes of brain regions produced at the later AD stages would help to improve the predication accuracy^45^.

This study still had some limitations. Firstly, the enrolled samples from the public data set were very limited, this increased the likelihood of individual influence during data collection and might have even led to overfitting during the predicted tasks. Then, since no other available public data sets were provided, the validation of our model would resort to private and multi-center data sets. Finally, only one type of imaging modality of sMRI was used in our study, and the application of multiple imaging modalities of MR and/or PET is still worthy of trying and evaluating.

In future, more cases with multi-modal data should be involved to improve the performance of predictions. In addition, more kinds of DL strategies such as transfer learning and reinforcement learning should be integrated to improve the accuracy, and reliability of the models for AD prediction^46,47^. Especially, the interpretability for proposed model would be investigated to improve the visualization and analysis of the features for the hidden layers.

## Materials and methods

### Materials

#### Enrolled participants

A total of 487 participants was enrolled from the Alzheimer’s disease neuroimaging initiative (ADNI) database (www.adni-info.org), including 111 patients with AD, 238 patients with MCI, and 138 normal controls. The subjects are primarily non-Hispanic white subjects. All enrolled participants met with the criteria of the national institute of neurological and communicative disorders and stroke/AD and related disorders association.

#### Data description

The data sets of sMRI, clinical scores (MMSE, ADAS-11, clinical dementia rating scale sum of boxes (CDRSB), and rey auditory verbal learning test (RAVLT) immediate, genetics (ApoE4), and biomarker features (Aβ42/Aβ40 antibodies, t-tau, and p-tau) at baseline were collected for each participant. And the demographic information of the enrolled participants is presented in Table 5. The chi-square tests validated that the variables such as sex, age, education years, MMSE, ADAS-11, CDRSB, and RAVLT immediate were individually independent and conformed to normal distribution among the three groups. Meanwhile, the clinical scores of MMSE and ADAS-11 at longitudinal time points of M06, M12, M24, and M36 were also collected for model evaluation, as shown in Table 6. The conversion cases of MCI groups were divided into two subgroups, pMCI and sMCI, as depicted in Table 7.

**Table 5:** The demographic information of enrolled subjects.

Groups	Gender (M/F)	Age	Education Years	MMSE	ADAS-11	CDRSB	RAVLT immediate
NC	69/69	73.98 ± 5.89	16.47 ± 2.61	28.99 ± 1.24	5.93 ± 3.03	0.03 ± 0.14	45.15 ± 9.99
MCI	137/101	75.18 ± 7.41	15.97 ± 2.59	28.19 ± 1.63	9.12 ± 4.26	1.39 ± 0.87	37.02 ± 10.60
AD	67/44	74.28 ± 8.41	15.58 ± 2.63	23.23 ± 2.04	21.05 ± 7.00	4.59 ± 1.60	21.57 ± 6.99

MMSE, mini-mental state examination; ADAS-11, AD assessment scale-cognitive 11-item; CDRSB, clinical dementia rating scale sum of boxes; RAVLT, rey auditory verbal learning test; NC, normal control; MCI, mild cognitive impairment; AD, Alzheimer’s disease.

**Table 6:** Clinical scores cases at different time points.

Clinical scores	Baseline	M06	M12	M24	M36
MMSE	487	458	439	357	199
ADAS-11	487	458	439	357	199

MMSE, mini-mental state examination; ADAS-11, AD assessment scale-cognitive 11-item.

**Table 7:** AD conversion cases in MCI group.

MCI stages	Baseline	M06	M12	M24	M36
pMCI	–	–	34	104	125
sMCI	238	238	204	134	113

pMCI, progressive MCI; sMCI, stable MCI; –, missed data.

All participants underwent MR scanning of the brain, which were acquired using 3 T scanners from Siemens, general electric (GE), and Philips at multiple sites. T1-weighted images were acquired, and the protocol parameters were listed as follows: 1.2 mm slice thickness; 256 × 256 scanning matrix; repetition time = 2300 ms; echo time = 2.98 ms; field of view = 240 × 240 mm^2^; flip angle = 90 degree; and 256 × 256 reconstruction matrix.

### Methods

**Figure 2:**
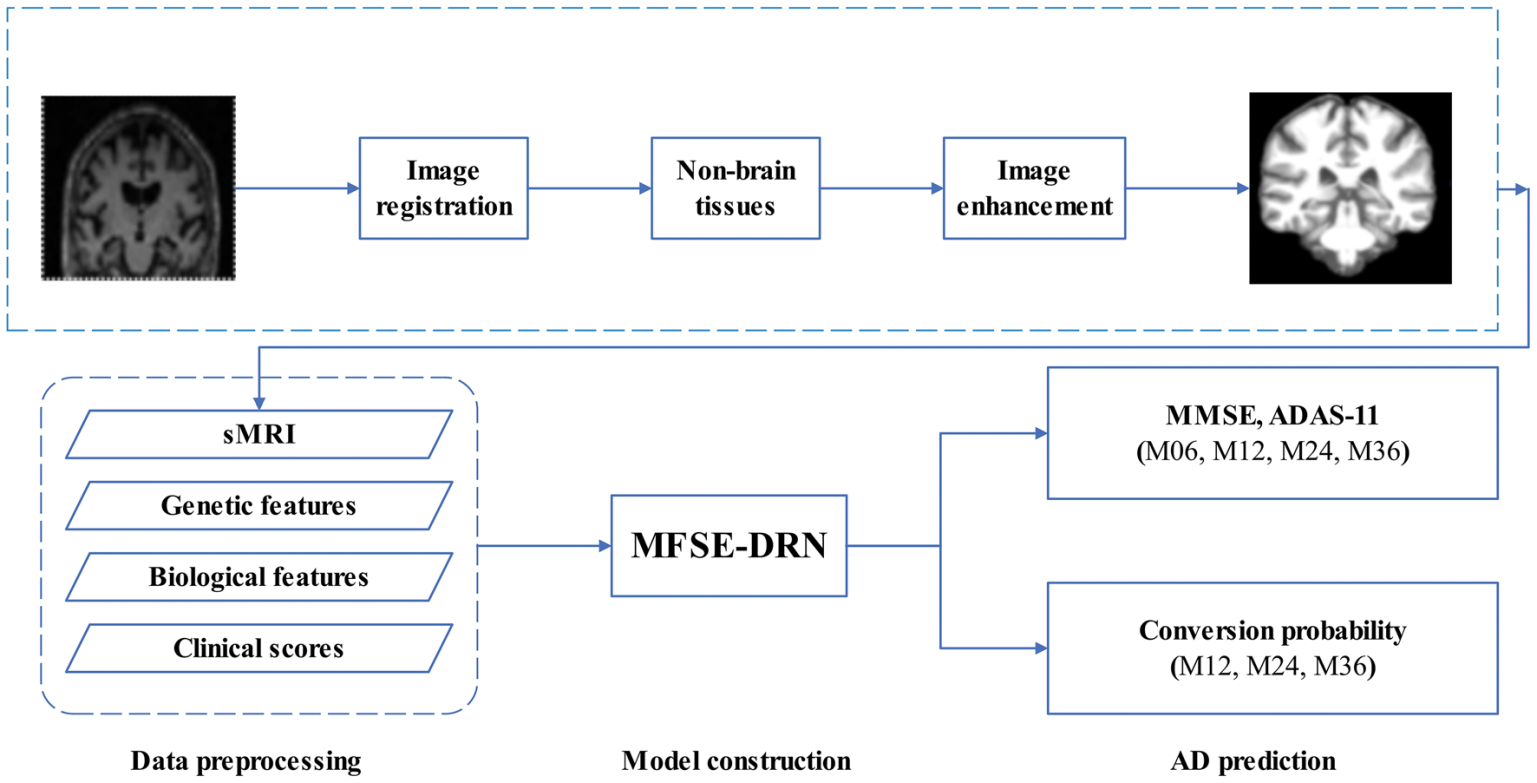
The pipeline of AD predication. sMRI: structural magnetic resonance imaging, MFSE-DRN: multifeature squeeze-and-excitation-dilated residual network, MMSE: mini-mental state examination, ADAS-11: AD assessment scale-cognitive 11-item.

The pipeline of the multifeature AD prediction included three steps: data preprocessing, model construction, and experimental setup (Fig. 2). Firstly, sMRI, genetic features, biological features and clinical information were individually preprocessed; secondly, the MFSE-DRN using the blocks of SE-DRB, MF-Pool and multi-feature fusion was constructed; and finally, the clinical scores and AD conversion probabilities were predicted.

### Data preprocessing

The data prepossessing contained two aspects: one for the sMRI data set, and another for clinical scores, genetic and biomarker data sets. The sMRI data preprocessing consisted of three steps: image registration, non-brain tissue segmentation, and image enhancement. It was performed using the computational anatomy toolbox 12 (CAT12) software package (https://www.nitrc.org/projects/cat/). Firstly, the sMRI images were registered using the diffeomorphic anatomical registration through exponentiated lie algebra (DARTEL) method to ensure brain structure alignment across individuals. Secondly, non-brain tissues, such as the skull and scalp, were removed using morphology-based methods. Thirdly, the image enhancement was achieved through histogram equalization, spatial filtering, and nonlinear transformation methods. Besides, the raw data sets of clinical scores, genes, and biomarkers were normalized due to the varied magnitude scales.

### Model construction of MFSE-DRN

**Figure 3:**
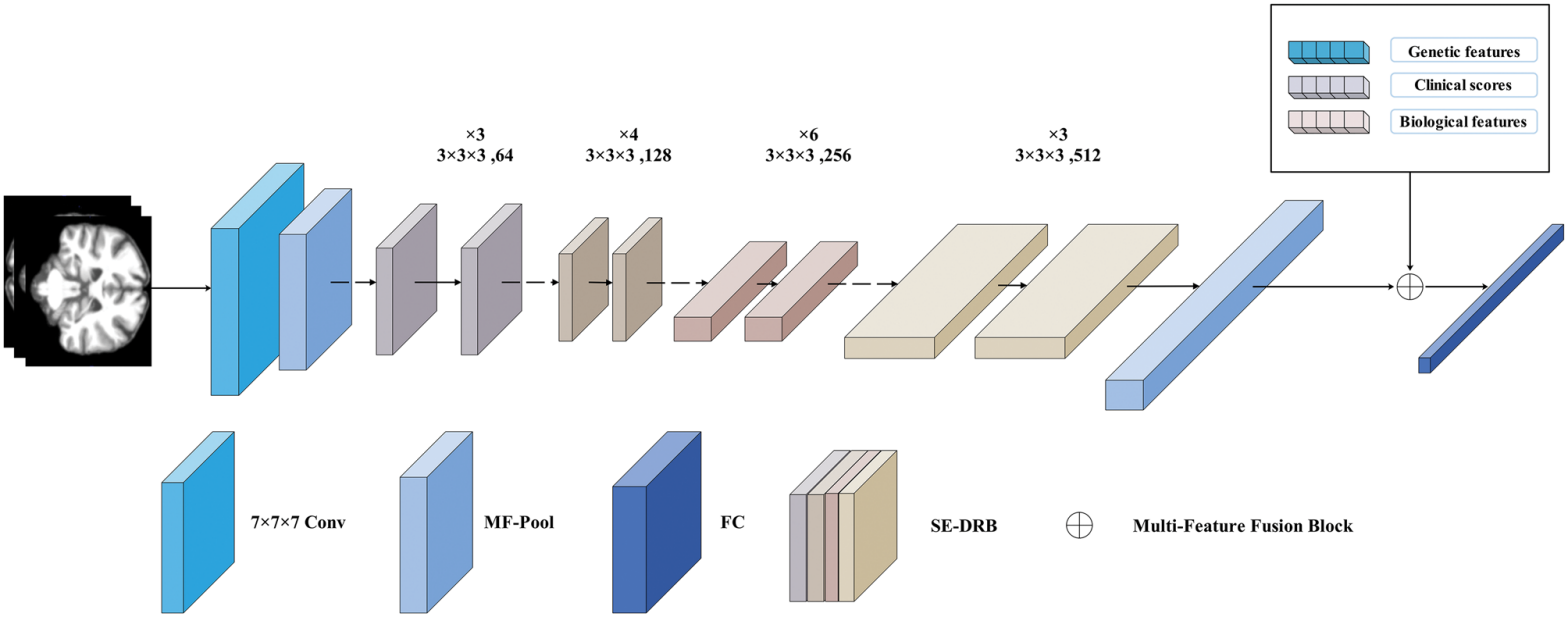
The architecture of MFSE-DRN. The solid lines indicate unchanged number of input and output channels, while the dashed lines denote changed number of input and output channels for short connections. MFSE-DRN: multifeature squeeze-and-excitation-dilated residual network, MF-Pool: multifusion pooling, FC: fully connected, SE-DRB: squeeze-and-excitation-dilated residual block.

Based on the classical residual network (DRN), our modified model integrated three key modules of SE-DRB, MF-Pool, and multifeature fusion. As illustrated in Fig. 3, our model comprised of 36 layers, including 32 convolutional (Conv) layers (16 SE-DRBs), one 7 × 7 × 7 Conv layer, 2 MF-Pool layers, and one multifeature fusion layer. The data pipeline of MFSE-DRN is represented as pseudo-code, as shown in Table 8.

**Table 8:**
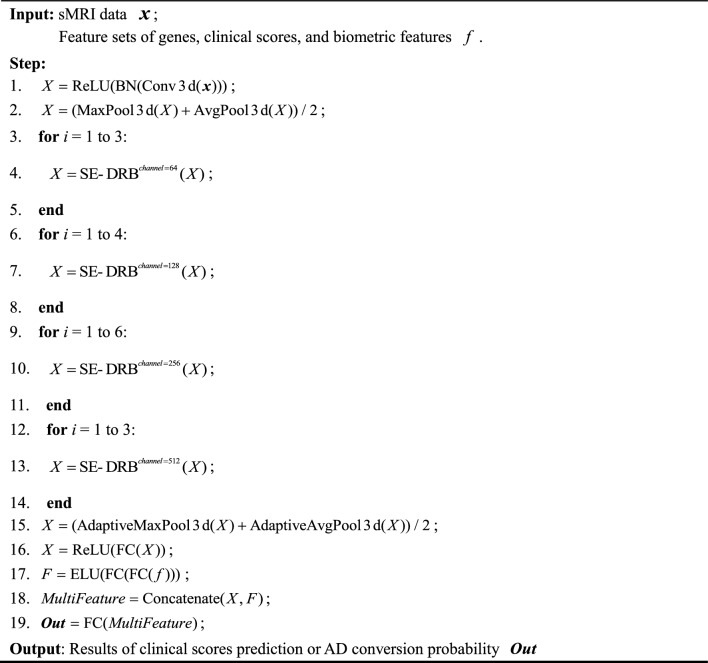
MFSE-DRN algorithm.

The SE-DRB was assembled by dilated convolution (DC) and squeeze-and-excitation (SE) blocks and enlarged the receptive convolution field for feature extraction. Then, the MF-Pool combined the max pooling and average pooling layers for extracting varying dimensions features. Furthermore, the multifeature fusion could effectively handle high-dimensional data. It concatenated concurrent features different levels of multiple features to extract the most representative features, and utilized the correlation and complementarity between different features to improve the accuracy of prediction.

The pipeline of our model was elucidated as follows: Firstly, the sMRI data was downsampled using a 7 × 7 × 7 Conv layer and a 3 × 3 × 3 MF-Pool layer. Next, the high-dimensional image features were extracted by the 16 SE-DRBs and outputted to the MF-Pool layer. At last, the image features were integrated with genetic features, clinical scores, and biological features, and the multi-features were connected with the fully connected (FC) layer. Since the proposed MF-Pool combined the max pooling and average pooling layers, and the capability of feature extraction at varied dimensions was effectively enhanced^48^. The final output of the MF-Pool is formulated as follows:

1 $$Y\left( {n,c,h,\omega } \right) = \frac{1}{2}\left[ {Y_{1} \left( {n,c,h,\omega } \right) + Y_{2} \left( {n,c,h,\omega } \right)} \right]$$

2 $$Y_{1} \left( {n,c,h,\omega } \right) = \mathop {max}\limits_{{m \in \left[ {0,k_{h} - 1\left] {,n \in } \right[0,k_{\omega } - 1} \right]}} X\left( {n,c,hs + m,\omega s + n} \right)$$

3 $$Y_{2} \left( {n,c,h,\omega } \right) = \frac{1}{{k_{h} \times k_{\omega } }}\mathop \sum \limits_{m = 0}^{{k_{h} - 1}} \mathop \sum \limits_{n = 0}^{{k_{\omega } - 1}} X\left( {n,c,hs + m,ws + n} \right)$$

where *Y* is the final output of the MF-Pool; *Y*_1_ is the output of the maximum pooling layer; *Y*_2_ is the output of the average pooling layer; *n* is the number of feature maps; *c* is the number of channels; *h* is the number of rows; *ω* is the number of columns; *s* is the step size; *k*_h_ is the pooling window length; *k*_ω_ is the pooling window width.

With the improved DRB, it facilitated the feature extraction capability for additional features by enlarging the Conv receptive field in residual networks. In our model, the DRB was constructed by replacing the first layer of the base convolution in the base residual module. Here, the base convolution kernel *k* = 3, DC expansion rate *d* = 2, equivalent convolution kernel *k’* = 5, and current sensory field *RF*_i+1_ = 7.

**Figure 4:**
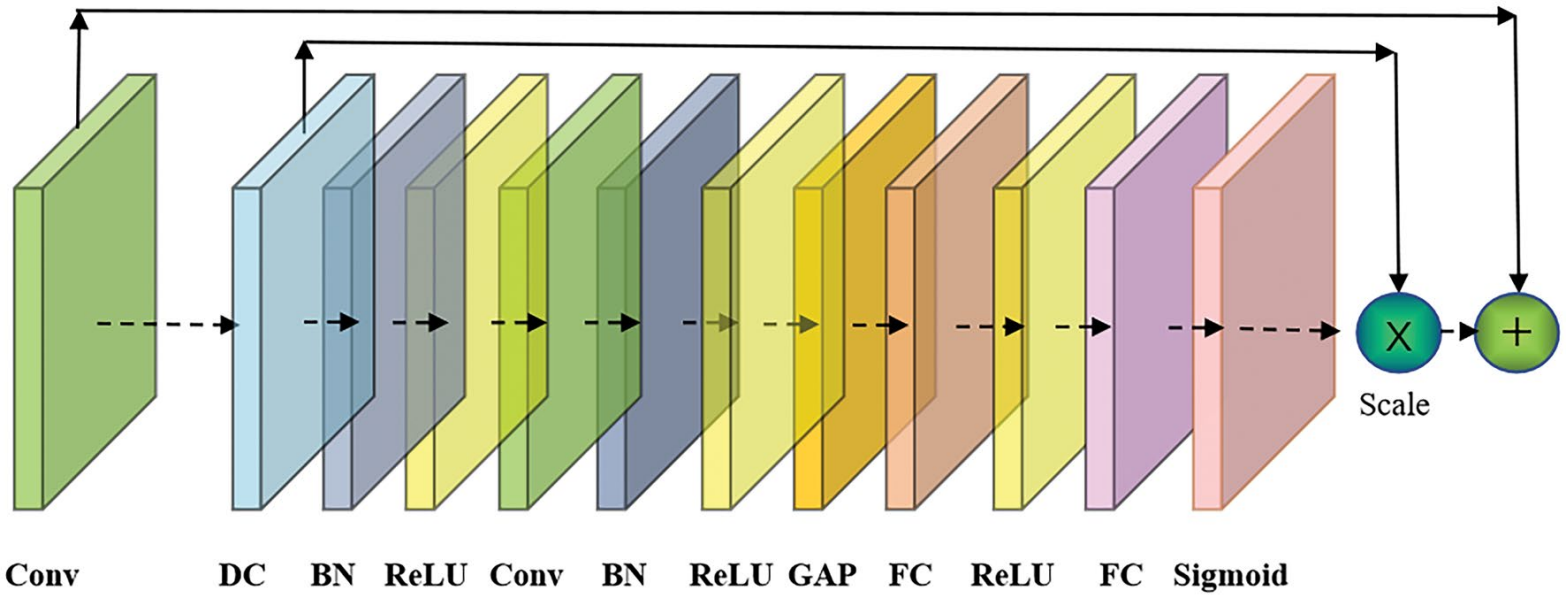
The diagram of SE-DRB layer. Conv: convolutional, DC: dilated convolution, BN: batch normalization, ReLU: rectified linear unit, GAP: global average pooling, FC: fully connected.

Additionally, the use of residual structure in our network architecture can allow for deeper and more complex models to be trained, and the short-circuit connection can effectively avoid the overfitting problem caused by the model depth^49^. In order to improve the model stability, the SE-DRB was built by integrating the SE with the DRB, as illustrated in Fig. 4. This made a more precise capture of feature correlations by assigning adaptive weights to features across various channels, thus significantly elevating the network performance.

**Figure 5:**
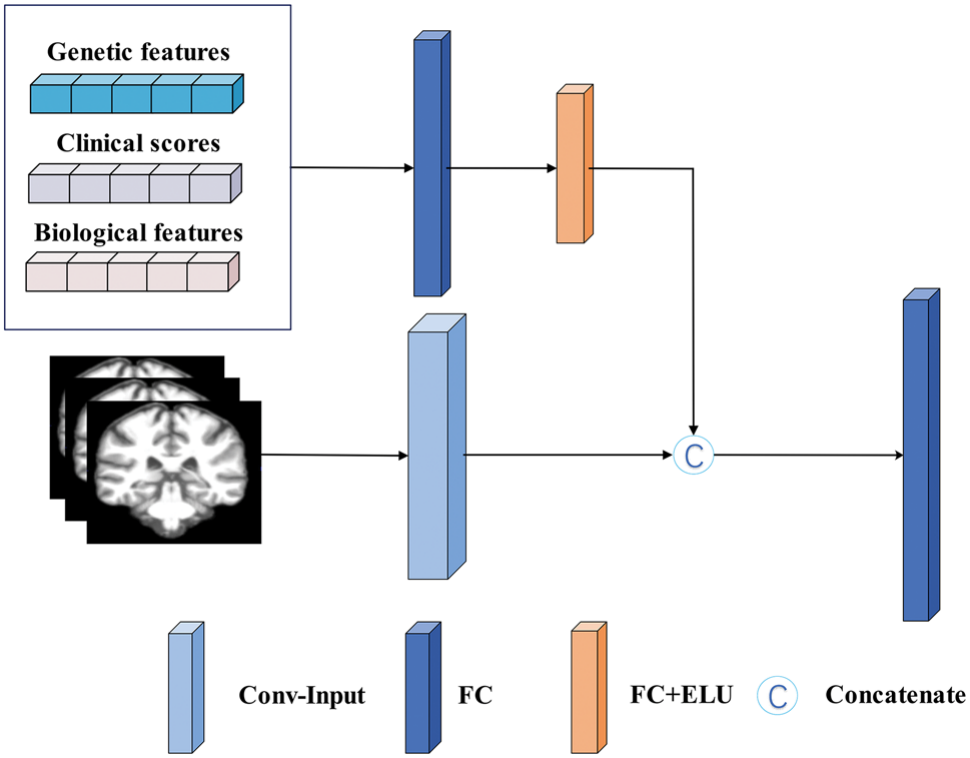
The chart of multi-feature fusion layer. Conv: convolutional, ELU: exponential linear unit, FC: fully connected.

In Fig. 5, the multimodal feature fusion block combined the image features extracted from the Conv layer with the genes, clinical scores, and biometric features, and acted on the FC layer to effectively extract multifeature information to achieve longitudinal prediction of AD.

### Experimental setup

In this study, two AD predicted tasks of clinical scores and conversion probability were performed. The first was to predict the MMSE and ADAS-11 scores at M06, M12, M24, and M36, and the second task was to predict the conversion probability of patients with MCI progressed to AD at M12, M24, and M36, respectively.

For performance evaluation, our proposed model was quantitatively compared with two other novel models: ranking convolutional neural network (RCNN) and 3D vision geometrical group network (3D-VGGNet)^50,51^. The ablation experiments were conducted by individually removing the three mentioned modules in the model, and five-fold cross-validation method with repeated random sampling is used.

During the experiments, The main hyperparameters were as follows: (1) during the clinical scores prediction, the number of training rounds was 150, the batch size was 8, the learning rate was 0.0001, the loss function was mean square error (MSE), and the optimizer was chosen as Adam; and (2) at the AD conversion probability prediction, the number of training rounds was 100, the batch size was 4, the learning rate was 0.0001, the loss function was Cross Entropy, and the Adam was chosen as optimizer. The regularization terms of L1 and L2 were implemented. Here, L1 produces a sparse matrix of weights and L2 allows the weights to decrease uniformly. This would ensure the model stability and avoid model overfitting.

Moreover, two metrics of MAE and RMSE were chosen for evaluating clinical scores prediction^52^, and five indices of ACC, precision, recall, F1, receiver operating characteristic, and AUC^53^ were defined to assess AD conversion probability prediction^54^.

The DL experiments were performed on a Windows 10 Pro workstation using an NVIDIA RTX 6000 graphics card. The CUDA version 11.3 and Python version 3.8 software were used with the DL framework of PyTorch.

### Ethical approval

The present study was approved by The Shanghai University of Medicine and Health Sciences ethics review committee (approval number, 2019-GZR-06-142132197606243519). All research methods were conducted in strict accordance with relevant guidlines and regulations. We hereby confirm that informed consent was obtained from all subjects and/or their legal guardians who provided data.

## Data Availability

The datasets generated or analyzed during the study are available in the Alzheimer’s Disease Neuroimaging Initiative (ADNI) repository, https://adni.loni.usc.edu/.
